# The role of trade and investment liberalization in the sugar-sweetened carbonated beverages market: a natural experiment contrasting Vietnam and the Philippines

**DOI:** 10.1186/s12992-015-0127-7

**Published:** 2015-10-12

**Authors:** Ashley Schram, Ronald Labonte, Phillip Baker, Sharon Friel, Aaron Reeves, David Stuckler

**Affiliations:** School of Epidemiology, Public Health and Preventative Medicine, University of Ottawa, 850 Peter Morand Crescent, K1G 5Z3 Ottawa, Canada; Regulatory Institutions Network (RegNet), Australian National University, H.C. Coombs Extension Building #8, Fellows Road, ACT 0200 Canberra, Australia; Department of Sociology, University of Oxford, Manor Road Building, Manor Road, OX1 3UQ Oxford, UK

**Keywords:** Trade and investment liberalization, Foreign direct investment, Sugar-sweetened carbonated beverages, Transnational companies, Natural experiment

## Abstract

**Background:**

Trade and investment liberalization may facilitate the spread of sugar-sweetened carbonated beverages (SSCBs), products associated with increased risk factors for obesity, type II diabetes, and cardiovascular diseases (Circulation 121:1356–1364, 2010). Apart from a limited set of comparative cross-national studies, the majority of analyses linking liberalization and the food environment have drawn on case studies and descriptive accounts. The current failure of many countries to reverse the obesity epidemic calls for investigation into both individual and systemic factors, including trade and investment policies.

**Methods:**

Using a natural experimental design we tested whether Vietnam’s removal of restrictions on foreign direct investment (FDI) subsequent to its accession to the World Trade Organization in 2007 increased sales of SSCBs compared with a matched country, the Philippines, which acceded in 1995. Difference-in-difference (DID) models were used to test pre/post differences in total SSCB sales and foreign company penetration covering the years 1999–2013.

**Results:**

Following Vietnam’s removal of restrictions on FDI, the growth rate of SSCB sales increased to 12.1 % per capita per year from a prior growth rate of 3.3 %. SSCB sales per capita rose significantly faster pre- and post-intervention in Vietnam compared with the control country the Philippines (DID: 4.6 L per annum, 95 % CI: 3.8 to 5.4 L, *p* < 0.008). Vietnam’s increase in SSCBs was primarily attributable to products manufactured by foreign companies, whose annual sales growth rates rose from 6.7 to 23.1 %, again unmatched within the Philippines over this period (DID: 12.3 %, 95 % CI: 8.6 to 16.0 %, *p* < 0.049).

**Conclusions:**

Growth of SSCB sales in Vietnam, led by foreign-owned companies, significantly accelerated after trade and investment liberalization.

## Background

There are growing concerns that liberalized trade and investment agreements create market conditions that facilitate the availability, sales, and consumption of unhealthy dietary products in low- and middle-income countries [1–3]. Rising consumption of sugar-sweetened beverages is particularly concerning given the body of epidemiological evidence linking consumption to obesity, type II diabetes, and cardiovascular diseases [4–6]. In children, each additional serving of a sugar-sweetened drink daily was associated with a 0.24 kg/m^2^ increase in body mass index and a 1.6 times greater odds of being obese, after adjusting for anthropometric, demographic, dietary, and lifestyle variables [7]. 21^st^ century trade agreements are increasingly used to open markets to foreign companies, expand investor protections, and privatize state-owned assets [8, 9]. Between 1995 and 2015, a total of 160 countries joined the World Trade Organization (WTO), signing up to trade agreements that, alongside a proliferation of bilateral and regional treaties, have opened markets to entry of foreign-owned food and beverage companies.

Increased trade and investment between nations may have positive health impacts. It can stimulate economic growth, potentially reducing poverty and its detrimental health impacts, promote investments in health care, education, and other population health determinants, and increase access to life-saving goods and technologies [10–12]. However, such health gains are not automatic and depend on progressive public policy for equitable distribution throughout society. There are potential health risks with trade and investment liberalization [13], including strong theoretical reasons to believe that trade and investment liberalization will lead to the spread of sugar-sweetened carbonated beverages (SSCBs) and other unhealthy dietary products through increased imports, foreign direct investment, and advertising [2, 14]. Yet few studies have been able to provide quantitative relational evidence of these effects.

Stuckler and colleagues evaluated exposure to US Free Trade Agreements across 80 countries, finding that those nations with a free trade agreement with the United States had 63.4 % higher soft drink sales per capita than those that did not, after correcting for GDP and other macroeconomic confounders [15]. Another study attempted to empirically link liberalization to diet-related health outcomes, such as obesity, finding support for the impact of economic globalization over and above those accounted for by GDP and urbanization [16]. A cross-national study of 25 countries between 1999 and 2008 found market deregulation policies facilitated the spread of fast food outlets, which correlated with higher population mean body mass indices among high-income countries. Apart from this limited set of comparative cross-national studies, the bulk of analyses have drawn on case studies and descriptive accounts. One study examined data in Mexico pre- and post-North American Free Trade Agreement (NAFTA), identifying subsequent increases in US exports of corn, soybeans, sugar, snack foods, and meat products as well as increased investment in production, processing, and retailing, that led to convergence in Mexican and US food systems [17]. Another performed a similar analysis of Central America Free Trade Agreement (CAFTA), identifying that the agreement led to increased availability of meat, dairy and processed food products, promoted domestic meat production and increased investment in the processed food market [18]. Case studies of Pacific island countries also suggest that trade policies accelerate nutrition transition [19, 20].

Obesity and diabetes continue to be pressing public health concerns, accounting for 2.8 and 1.5 million deaths globally each year, respectively [21]. To our knowledge, no country has reversed its obesity epidemic [22], suggesting that current approaches are inadequate. Conceptualizing and addressing the role of structural drivers of diet-related health outcomes, including trade and investment policy, is an important development in tackling the complexity of the problem. Two broadly differing frameworks have defined public health interventions addressing obesity. The individualizing framework, both more pervasive and market-friendly, places the onus on individuals and their ‘lifestyle’ choices, with little to no government regulatory action concerning the food industry. The systemic framework puts the onus on wider environmental factors and encourages governments to act on behalf of the public, including regulating food markets from production through to consumption [22]. This paper attempts to unpack some of the complexity at the systemic level by examining the role of trade and investment in the creation and maintenance of obesogenic food environments.

Key actors in the creation of food environments are transnational food and beverage corporations, companies like Coca-Cola and PepsiCo, which tend to dominate the soft drink industry in newly liberalized countries. Their financial positions allow them to invest in aggressive advertising campaigns with celebrity endorsements, and to utilize strategic partnerships with retail distributors and major consumer foodservice chains [23]. In 2013, sales of Coca-Cola and PepsiCo alone accounted for 68.7 % of the global carbonated beverage market [24]. As markets for SSCBs have become saturated in high-income countries [15], multi-nationals face pressure to identify emerging markets for growth. In the next five years, PepsiCo and Coca-Cola project their main source of growth in profits will come from developing countries [25, 26].

The impetus for the current analysis was to explore the impacts of previously ratified trade and investment treaties within vulnerable nations of the Trans-Pacific Partnership (TPP) agreement. At present, 12 Pacific Rim countries are negotiating what is thought to be the most economically significant preferential trade and investment agreement in history, representing a market of 792 million people and 40 % of global GDP [27]. States negotiating the TPP are economically, geographically, and demographically diverse; with GDP per capita (PPP) ranging from US $4000 in Vietnam to over $62,000 in Singapore [28, 29]. Vietnam is an especially vulnerable country involved in the treaty negotiations, with a GDP per capita over seven thousand dollars less than the next economically weakest member, Peru [30].

Vietnam’s membership in the TPP negotiations places it at risk for a number of domestic policy changes and regulatory restructuring based upon known or anticipated content of the proposed treaty [31]. One of the controversial elements of the treaty is the inclusion of investor-state dispute settlement (ISDS) mechanisms, details of which have already become public in leaked draft texts of the TPP agreement. ISDS allows foreign investors to sue national governments when they feel their investment has been expropriated due to government actions, including the ability to seek financial recourse against state actions addressing public welfare that may unfavorably affect their investment. This has many in public health concerned for the viability of introducing new regulations to control the influx of processed food and beverages [32, 33], particularly among resource-constrained developing countries that are key markets for such products. Vietnam is one of the few countries that does not currently have any ISDS mechanisms in place, thus signing the TPP with ISDS would represents a new vulnerability to which it has not previously been exposed. This vulnerability becomes more evident when we consider the effects of recent trade and investment liberalization on the food environment in Vietnam, since ISDS provisions in the TPP could make it difficult for it to introduce new regulations to govern said environment for public health purposes.

In this paper we test the hypothesis that Vietnam’s trade liberalization resulting from WTO membership would lead to a significant increase in SSCB sales, particularly among foreign companies (namely Coca-Cola and PepsiCo), contrasted alongside the experience of the Philippines. Accession to the WTO involves a comprehensive set of commitments, obligations and enforcement measures requiring considerable reconstruction of domestic policies generally perceived to reduce the role of government in markets while increasingly privatizing the production and distribution of goods and services [34]. According to the World Bank the cost of accession is rising, with higher levels of liberalization expected from new members [35]. Our study is intended to sit alongside a similar analytical approach focusing on Peru, the second ‘least wealthy’ TPP nation [36]. These two papers contribute to the body of quantitative evidence exploring the diet-related health effects of trade and investment agreements by providing robust evidence for the link between investment liberalization and changes to the food environment, namely SSCBs. The findings have implications for how increased trade and investment liberalization commitments in the TPP will likely continue affecting health-harmful diet-related changes, and should be utilized by health and trade ministries to make informed policy decisions.

## Methods

### Study design and case selection

We employed a ‘natural experiment’ design, which takes advantage of variations in the timing, geography, or eligibility of an intervention. These are recommended in situations when randomized trials are not available for ethical or pragmatic reasons, as is the case with trade treaties [37]. Unlike in randomized controlled trials, in a natural experiment the intervention is assigned by a policy or other exogenous socio-environmental change, not by the researcher.

**Fig. 1:**
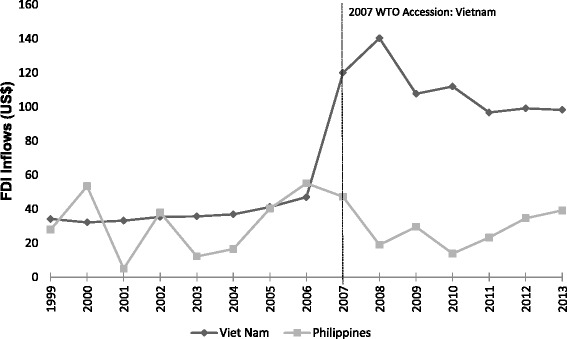
Trends in FDI Inflows in Vietnam and the Philippines before and after Vietnam’s 2007 WTO accession

A natural experiment occurred on 11 January 2007 when Vietnam joined the WTO. As part of the agreement, Vietnam began a process of liberalizing its markets to allow greater entry by foreign owned companies through foreign direct investment (FDI); although market access commitments specific to SSCBs were only fully implemented as of 2009 (see Table 1 for a detailed account of these commitments). The impacts of Vietnam’s WTO accession may have been enhanced by a bilateral agreement it entered into with the US in 2001 which largely paralleled its WTO commitments, permitting US companies access to services relevant to the beverage sector just weeks before remaining WTO members. As shown in Fig. 1, there was a substantial entry of FDI into Vietnam post WTO accession. Prior to entry, from 1999 to 2006, FDI flows averaged about US $37.0 per capita annually. Following the trade agreement, the average flow rose to US $110.6 per capita annually in the years 2007 to 2013. While it is not possible to obtain a detailed sectoral breakdown, in 2013 manufacture and processing accounted for 56 % of the value of these FDI inflows, while warehouse and transportation, and wholesale, retail and maintenance services each captured 2 %; FDI from the beverage industry could potentially be counted in all of the aforementioned sectors. Currently Vietnam is projected to be one of the largest growth markets for Coca-Cola and PepsiCo over the next few years [25, 26].

**Table 1:** Comparing trade and investment liberalization in Vietnam and the Philippines

Foreign direct investment (FDI) can be facilitated through three different policy measures: (1) multilateral liberalization commitments under mode 3 (commercial presence) in the WTO General Agreement on Trade in Services (GATS); (2) bilateral or regional liberalization commitments; and (3) unilateral liberalization commitments made by governments outside of binding trade and investment treaty commitments. Four economic sectors are relevant to investments in the SSCB market: wholesale and retail distribution; services incidental to manufacturing; advertising services; and market research services. The term liberalization used throughout the paper refers collectively to these sectors.
The following provides a brief overview of the trade and investment liberalization strategies of Vietnam and the Philippines, including a review of the identified service sector commitments at the bilateral (with the US), multilateral (WTO), and unilateral (domestic) levels.
Vietnam
*Bilateral relations with the United States*: In 1975, as a result of the victory of the communist party of North Vietnam over the US-backed anti-communist party of South Vietnam, the US severed economic relations with the country until 1994, when the 19 year long trade embargo was lifted. On 10 December 2001, Vietnam and the US entered into a bilateral trade agreement which would permit 100 % US invested capital into wholesale and retail services and unlimited capital contributions on US joint ventures in advertising and market research services seven years after entry [58]. Effectively, by 10 December 2008 services relevant to US beverage companies had been fully liberalized, with the exception that services incidental to manufacturing were not liberalized through this agreement. In 2007 the two nations signed a Trade and Investment Framework Agreement to establish a formal dialogue to discuss new initiatives to deepen the trade and investment relationship. Currently, the US and Vietnam are both negotiating members of the Trans-Pacific Partnership Agreement.
*Multilateral liberalization*: After twelve years of preparation, Vietnam formally acceded to the WTO on 11 January 2007. Membership in the WTO required numerous changes to its legal and regulatory framework regarding taxation, intellectual property, price and foreign exchange controls, as well as enactment of the Law on Investment and the Law on Enterprises, both of which made domestic and foreign investors subject to the same laws and put them on more equal terms [59]. Accession to the WTO liberalized 105 service sectors in Vietnam, although progressive liberalization was built into some sectors [60]. Vietnam’s commitments in the General Agreement on Trade in Services (GATS) paralleled many of those in the US bilateral agreement, such that 100 % foreign invested capital into wholesale and retail services and unlimited foreign capital in joint ventures in advertising and market research services would be permitted by 1 January 2009, just weeks after the US-bilateral commitments took effect. Additionally, their WTO commitments included services incidental to manufacturing, though not taking effect until 1 January 2010, and limited to joint ventures with foreign capital contribution not exceeding 50 %.
*Unilateral liberalization*: Countries can unilaterally liberalize foreign investment in their economies outside of trade treaty commitments. Since Vietnam has ‘locked in’ their foreign investment polices at the multilateral and bilateral levels, however, a deeper exploration of its unilateral domestic policies on foreign investment liberalization is unnecessary for our purposes.
The Philippines
*Bilateral relations with the United States*: On 4 July 1946 the US granted the Philippines its independence, 48 years after it had been ceded the archipelago by the Spanish for the amount of $US 20 million. The US and the Philippines have had uninterrupted economic relations for more than a hundred years and, although they signed a Trade and Investment Framework Agreement (TIFA) in November 1989, no bilateral trade agreements or investment treaties have been produced. The Philippines is not currently a negotiating member of the TPP, although they have expressed strong interest in joining [61] out of fear of losing their share of the US market to participating neighboring countries and have been involved in technical consultations with the United States Trade Representative (USTR) [62].
*Multilateral liberalization*: The Philippines acceded to the WTO upon its inception on 1 January 1995. The Philippines liberalized 51 service sectors through the GATS [60]. Notably, their GATS commitments do not include any guaranteed market access for manufacturing, wholesale or retail, advertising, or market research services.
*Unilateral liberalization*: The Philippines went through an accelerated stage of FDI liberalization domestically in 1991 with their Foreign Investment Act [63], which permitted foreign equity up to 100 % in any sector not specified in the Foreign Investment Negative List (FINL). The FINL originally included a section that restricted investment in adequately served sectors, where no further investments were thought necessary, but this was abolished in 1996. As of 2012 the FINL does not specify any limitations on the manufacture or wholesale distribution of food and beverages, or market research services, though foreign equity in advertising is limited to 30 %. Retail trade services, previously limited to Filipino nationals, was liberalized in March 2000 through the Retail Trade Liberalization Act [64], which allows foreign investors 100 % ownership of retail business pending a minimum of US$7.5 million in equity.
Contrasting approaches
Vietnam and the Philippines have had considerably different approaches to trade and investment liberalization. The Philippines engaged with the global market much earlier on having long-term economic relations with the US and joining the WTO upon its creation, but has been quite stagnant since then. Vietnam, as a former closed economy with strained US relations, was previously relatively inactive in the global economy. However, as of late it has taken an aggressive approach to opening its borders to trade and investment, such that Vietnam has opened twice as many service sectors as the Philippines through GATS, providing protected market access for these sectors into the future. Additionally, Vietnam’s liberalization has occurred at the multilateral level where there are international channels for settling disputes, unlike the Philippines unilateral liberalization which only provides domestic dispute procedures for investors.

To identify the impact of the trade agreement on SSCB sales we compare the intervention group, Vietnam, with a control group that was not similarly exposed but was similar in other respects. Here, neighboring country, the Philippines, serves as the control. It had early engagement in trade relations with the United States, joined the WTO in 1995, and did not experience a marked change in FDI from 1999 to 2013, but it has a similar demographic profile and GDP per capita as Vietnam ($4700 and $4000, respectively) [28, 38] (see also Table 1 for an overview of the Philippines trade and investment commitments). We also established a control product for SSCBs, specifically, an aggregate of unprocessed foods. These products served as a control, as previous research has demonstrated these areas are less likely to be targeted by FDI from transnational food and beverage companies since they have lower profit margins [3]. Finally, we were interested in SSCB sales growth specific to foreign companies, and utilized domestic company sales as a control variable.

### Statistical analysis

We performed four difference-in-difference (DID) models before and after the 2009 liberalization of SSCB market access commitments: testing differences in SSCBs between Vietnam and the Philippines (1); differences between SSCBs and unprocessed foods in Vietnam (2a) and in the Philippines (2b); differences in foreign company sales between Vietnam and the Philippines (3); and differences between foreign and domestic company sales in Vietnam (4a) and in the Philippines (4b). Our models were as follows; where T1 represents estimates in the pre-intervention period; T2 represents estimates in the post-intervention period; UPF represents unprocessed foods; and FCS and DCS represent foreign and domestic company sales, respectively:

1 $$ \Delta \Delta \mathrm{SS}\mathrm{C}\mathrm{B} = \left(\Delta \mathrm{SS}\mathrm{C}{\mathrm{B}}_{\mathrm{Vietnam}}\left[\mathrm{S}\mathrm{S}\mathrm{C}{\mathrm{B}}_{\mathrm{T}2} - \mathrm{S}\mathrm{S}\mathrm{C}{\mathrm{B}}_{\mathrm{T}1}\right]\hbox{--}\ \Delta \mathrm{SS}\mathrm{C}{\mathrm{B}}_{\mathrm{Philippines}}\left[\mathrm{S}\mathrm{S}\mathrm{C}{\mathrm{B}}_{\mathrm{T}2}\hbox{--}\ \mathrm{S}\mathrm{S}\mathrm{C}{\mathrm{B}}_{\mathrm{T}1}\right]\right) $$

2a $$ \Delta \Delta \mathrm{SSCB}/\mathrm{UP}{\mathrm{F}}_{\mathrm{Philippines}} = \left(\Delta \mathrm{SSCB}\ \left[\mathrm{S}\mathrm{S}\mathrm{C}{\mathrm{B}}_{\mathrm{T}2}\hbox{--}\ \mathrm{S}\mathrm{S}\mathrm{C}{\mathrm{B}}_{\mathrm{T}1}\right]\ \hbox{--}\ \Delta \mathrm{UPF}\ \left[\mathrm{UP}{\mathrm{F}}_{\mathrm{T}2}\hbox{--}\ \mathrm{UP}{\mathrm{F}}_{\mathrm{T}1}\right]\right) $$

2b $$ \Delta \Delta \mathrm{SSCB}/\mathrm{UP}{\mathrm{F}}_{\mathrm{Philippines}} = \left(\Delta \mathrm{SSCB}\ \left[\mathrm{S}\mathrm{S}\mathrm{C}{\mathrm{B}}_{\mathrm{T}2}\hbox{--}\ \mathrm{S}\mathrm{S}\mathrm{C}{\mathrm{B}}_{\mathrm{T}1}\right]\ \hbox{--}\ \Delta \mathrm{UPF}\ \left[\mathrm{UP}{\mathrm{F}}_{\mathrm{T}2}\hbox{--}\ \mathrm{UP}{\mathrm{F}}_{\mathrm{T}1}\right]\right) $$

3 $$ \Delta \Delta \mathrm{F}\mathrm{C}\mathrm{S} = \left(\Delta \mathrm{F}\mathrm{C}{\mathrm{S}}_{\mathrm{Vietnam}}\left[\mathrm{F}\mathrm{C}{\mathrm{S}}_{\mathrm{T}2}\hbox{--}\ \mathrm{F}\mathrm{C}{\mathrm{S}}_{\mathrm{T}1}\right]\ \hbox{--}\ \Delta \mathrm{F}\mathrm{C}{\mathrm{S}}_{\mathrm{Philippines}}\left[\mathrm{F}\mathrm{C}{\mathrm{S}}_{\mathrm{T}2}\hbox{--}\ \mathrm{F}\mathrm{C}{\mathrm{S}}_{\mathrm{T}1}\right]\right) $$

4a $$ \Delta \Delta \mathrm{FCS}/\mathrm{D}\mathrm{C}{\mathrm{S}}_{\mathrm{Vietnam}} = \left(\Delta \mathrm{FCS}\ \left[\mathrm{F}\mathrm{C}{\mathrm{S}}_{\mathrm{T}2}\hbox{--}\ \mathrm{F}\mathrm{C}{\mathrm{S}}_{\mathrm{T}1}\right]\ \hbox{--}\ \Delta \mathrm{DCS}\ \left[\mathrm{D}\mathrm{C}{\mathrm{S}}_{\mathrm{T}2}\hbox{--}\ \mathrm{D}\mathrm{C}{\mathrm{S}}_{\mathrm{T}1}\right]\right) $$

4b $$ \Delta \Delta \mathrm{FCS}/\mathrm{D}\mathrm{C}{\mathrm{S}}_{\mathrm{Philippines}} = \left(\Delta \mathrm{FCS}\ \left[\mathrm{F}\mathrm{C}{\mathrm{S}}_{\mathrm{T}2}\hbox{--}\ \mathrm{F}\mathrm{C}{\mathrm{S}}_{\mathrm{T}1}\right]\ \hbox{--}\ \Delta \mathrm{DCS}\ \left[\mathrm{D}\mathrm{C}{\mathrm{S}}_{\mathrm{T}2}\hbox{--}\ \mathrm{D}\mathrm{C}{\mathrm{S}}_{\mathrm{T}1}\right]\right) $$

The DID models utilized the average of annual per capita sales estimates over the pre- and post-intervention years. In order to detect changes in sales we time-lagged the intervention point one year after liberalization of the SSCB market access commitments to allow time for the effects of the new investment commitments to take place. Thus the intervention year is considered to be 2009 with the effects of the intervention beginning to take effect in 2010, making our pre-intervention period inclusive of the years 1999–2009, and the post-intervention period inclusive of the years 2010–2013 (with the exception of sales data by foreign and domestic companies, which were only available post 2004). We also conducted a series of sensitivity tests to see whether our results are robust to different model specification. Changes in sugar-sweetened beverages may have been linked to changes in economic growth. To test this relationship we adjusted our models for GDP, finding that our results did not qualitatively change. Next, we included a linear time trend in the model to test whether the observed increase in sugar-sweetened beverages is consistent with the background trend. The observed rise is so large that it is very unlikely to be explained by the pre-intervention data alone.

After an initial examination of the data it was decided that actual volumes were only applicable for use in the first test (comparing SSCB sales volumes between Vietnam and Philippines), while the remaining analyses would require growth rates to compensate for variability in the scales (i.e., contrasting volumes measured in litres (L) and tonnes, and when ranges of values were too large for comparison). All models were performed using STATA v13.0.

### Sources of data

Growth of SSCB sales data were taken from the Euromonitor Database 2014 edition in units of litres per capita sold off-trade (i.e., through retail outlets), covering the years 1999–2013. Euromonitor’s carbonated beverages category is inclusive of all sweetened (both naturally and artificially) non-alcoholic drinks containing carbon dioxide, including all carbonated products containing fruit juice (“sparkling juices”), but excludes tea-based drink, energy drinks and carbonated bottled water. It is important to note the variety of sweeteners that can be utilized. The first category is *nutritive sweeteners* or *caloric sweeteners*, which includes sucrose (sugar cane and sugar beets [normal table sugar] and its derivatives), as well as agave nectar, corn syrup, dextrose, fructose, glucose, high-fructose corn syrup, honey, inverted sugar, lactose, maple syrup, and molasses [39, 40]. Some sugars naturally occur in foods (e.g., fructose in fruit juices), while others (e.g., sucrose) are added sugars. The second category is *nonnutritive sweeteners* or *noncaloric sweeteners* including aspartame, sucralose, saccharin, stevia, acesulfame K, neotame, nectresse and cyclamates [40, 41].

Carbonated beverages can be sweetened with any combination of these sweeteners, although high-fructose corn syrup is the most common source according to US data [42]. In this article we aim specifically to explore sugar-sweetened carbonated beverages (i.e., nutritive or caloric sweeteners) given their link to diabetes and obesity. While Euromonitor does not disaggregate the data by caloric and noncaloric sweeteners, an examination of the SSCB market data between 2009 and 2014 by brand shares reveals that noncaloric or ‘diet’ brands comprise only 1.4 % of the market in Vietnam and 2.3 % of the market in the Philippines (data were unavailable before 2009). While it is not possible to remove these diet products from the aggregated data we believe that their contribution remains negligible.

Sales of unprocessed foods (i.e., excluding packaged and processed products) were based on aggregating sales data for fresh eggs, fruits, meats, nuts, seafood, and vegetables. We further disaggregated sales data into those attributable to foreign and domestic beverage companies.

## Results

### Comparing SSCBs in Vietnam and the Philippines

**Fig. 2:**
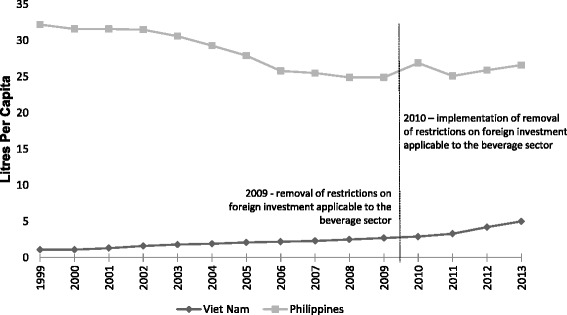
Trends in SSCB sales in Vietnam and the Philippines before and after Vietnam’s expanded liberalization commitments

Figure 2 shows the trends in SSCB sales in Vietnam and the Philippines before and after Vietnam’s implementation of FDI liberalization. Average per capita sales of SSCBs in Vietnam rose from 1.9 L (95 % CI: 1.6 to 2.2) to 3.9 L (95 % CI: 3.4 to 4.3) post-intervention. Over the same period per capita sales in the Philippines dropped from 28.7 L (95 % CI: 28.4 to 29.0) to 26.1 L (95 % CI: 25.6 to 26.6). The DID model revealed a significant difference between the two countries pre- and post-intervention (4.6 L, 95 % CI: 3.8 to 5.4, *p* = 0.008) that was robust to adjustments for GDP and underlying time trends (see Table 2).

**Table 2:** Pre and post 2010 SSCB sales differences between Vietnam and the Philippines

	Unadjusted	Adjusted for GDP	Adjusted for GDP and time trends
Between country	4.6**	4.6**	4.3**
Difference-in-difference estimate	(1.6)	(1.5)	(1.3)
R-squared	0.98	0.98	0.98

**p < 0.01

### Comparing SSCBs with unprocessed food in Vietnam and the Philippines

**Fig. 3:**
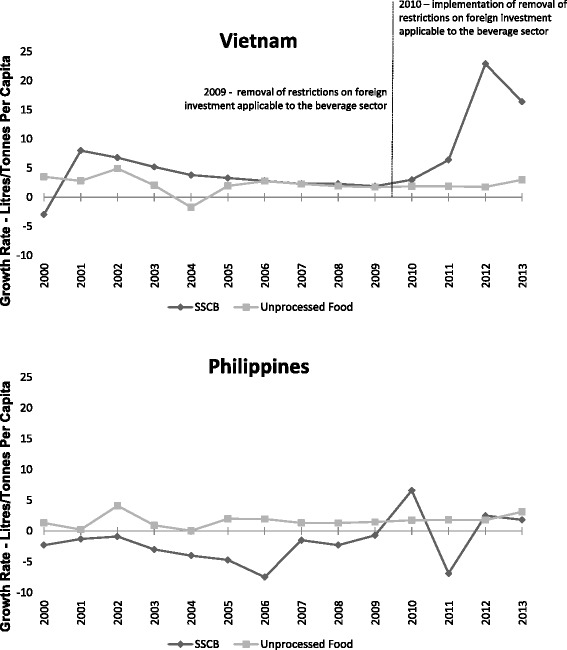
Trends in SSCB and unprocessed food sales in Vietnam and the Philippines, before and after Vietnam’s expanded liberalization commitments

Figure 3 displays the trends in sales growth of SSCBs and unprocessed food within Vietnam and the Philippines. There was substantial sales growth in SSCBs in Vietnam post-intervention, with a growth rate of 12.1 % (95 % CI: 11.1 to 13.1) relative to the prior growth rate of 3.3 % (95 % CI: 2.7 to 4.0); while sales growth in the unprocessed food category remained largely unaffected, with a post-intervention rate of 2.1 % (95 % CI: 1.1 to 3.1) and a 2.2 % growth rate prior (95 % CI: 1.6 to 2.9). This contrasts with the data shown for the Philippines, which equally had little movement in the growth rates of unprocessed food from pre-intervention (1.5 %; 95 % CI: 1.1 to 1.9) to post-intervention (2.1 %; 95 % CI: 1.5 to 2.8); but showed a tendency for negative growth rates in SSCB sales pre-intervention (−2.8 %; 95 % CI: −3.2 to −2.4); and no discernible trend towards increased growth post-intervention (1.0 %; 95 % CI: 0.4 to 1.7). The DID model supported a significant difference between the two categories in Vietnam (8.9 %; 95 % CI: 7.3 to 10.6, *p* = 0.011), robust to adjustment for GDP and underlying time trends, and no significant difference within the Philippines (3.2 %; 95 % CI: 2.1 to 4.3, *p* = 0.141, see Table 3).

**Table 3:** Pre and post 2010 differences in SSCB and unprocessed foods^a^ within Vietnam and the Philippines

	Vietnam			Philippines		
	Unadjusted	Adjusted for GDP	Adjusted for GDP and time trends	Unadjusted	Adjusted for GDP	Adjusted for GDP and time trends
Within country	8.9*	8.9*	8.9*	3.2	3.2	3.1
Difference-in-difference estimate	(3.2)	(3.2)	(3.4)	(2.1)	(2.1)	(2.2)
R-squared	0.47	0.47	0.47	0.45	0.45	0.45

*p < 0.05

^a^Aggregate of sales growth in tonnes of eggs, meats, seafood, fruits, vegetables and nuts

### Comparing foreign company sales growth in Vietnam and the Philippines

**Fig. 4:**
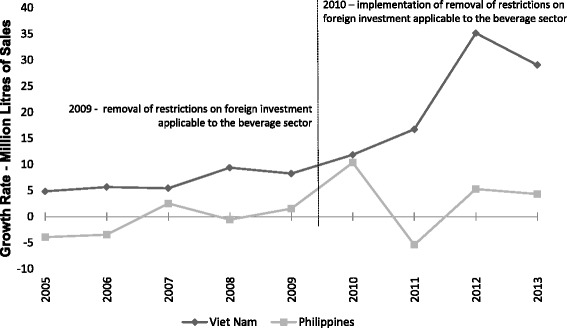
Trends in Foreign SSCB sales in Vietnam and the Philippines, before and after Vietnam’s expanded liberalization commitments

Figure 4 presents the trends in sales growth in millions of litres of SSCBs by foreign companies in Vietnam and the Philippines. Foreign sales growth rates in Vietnam rose rapidly post-intervention from 6.7 % (95 % CI: 4.9 to 8.5) annually to 23.1 % (95 % CI: 21.1 to 25.1), a level of growth unmatched in the Philippines, which showed a modest rise from −0.8 % (95 % CI: −2.58 to 1.0) annually to 3.6 % (1.6 to 5.7). The unadjusted DID model failed to find a significant difference (*p* = 0.057); although after adjusting for GDP and underlying time trends, the difference between the two countries differences pre- and post-intervention was significant (12.3 %; 95 % CI: 8.6 to 16.0, *p* = 0.049, see Table 4).

**Table 4:** Pre and post 2010 differences in foreign sales between Vietnam and the Philippines

	Unadjusted	Adjusted for GDP	Adjusted for GDP and time trends
Between country	12.1****	12.4*	12.3*
Difference-in-difference estimate	(5.8)	(5.4)	(5.6)
R-squared	0.73	0.78	0.78

*p < 0.05, ****p = 0.057

### Comparing foreign with domestic company sales growth in Vietnam and the Philippines

**Fig. 5:**
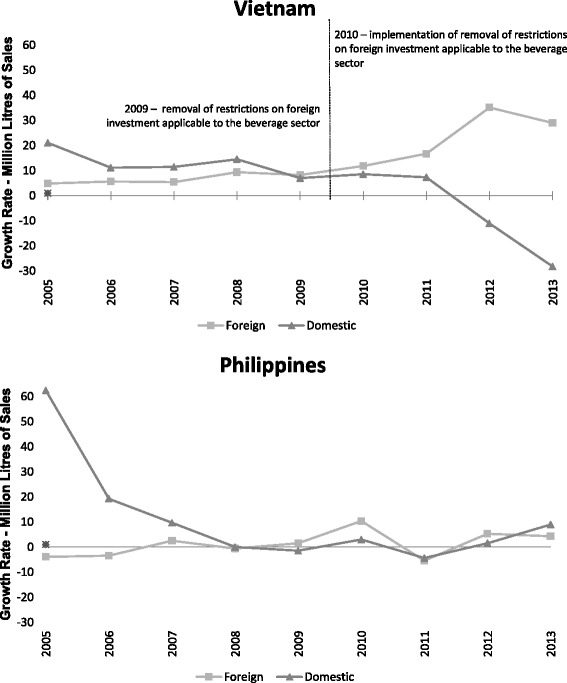
Trends in foreign and domestic SSCB Sales in Vietnam and the Philippines, before and after Vietnam’s expanded liberalization commitments

Trends over time in the sales growth of SSCBs in millions of litres for all foreign and domestic beverage companies for Vietnam and the Philippines are presented in Fig. 5. Sales growths for foreign companies in both countries are reported above. Sales growth for domestic companies declined in Vietnam, from 13.1 % (95 % CI: 10.2 to 16) annually to −5.8 % (95 % CI: −9.1 to −2.6) post-intervention. The Philippines also had a considerable decline in domestic sales growth over the same period, from 18.0 % (95 % CI: 15.1 to 20.9) annually to 2.3 % (95 % CI: −1.0 to 5.6). The DID model supported a significant difference between foreign and domestic sales growth in Vietnam (35.4 %; 95 % CI: 29.3 to 41.5, *p* = 0.002), robust to adjustment for GDP and underlying time trends, and no significant difference within the Philippines (20.1 %; 95 % CI: 11.0 to 29.2, *p* = 0.170, see Table 5).

**Table 5:** Pre and post 2010 differences between foreign and domestic sales within Vietnam and the Philippines

	Vietnam			Philippines		
	Unadjusted	Adjusted for GDP	Adjusted for GDP and time trends	Unadjusted	Adjusted for GDP	Adjusted for GDP and time trends
Difference-in-difference estimate	35.4**	35.0**	33.6**	20.1	19.6	21.7
	(9.4)	(9.2)	(8.85)	(13.9)	(13.7)	(13.1)
R-squared	0.56	0.61	0.68	0.25	0.33	0.44

**p < 0.01

### Foreign and domestic company concentration in Vietnam and the Philippines

Sales of SSCBs in the Philippines are more heavily concentrated within foreign companies (98.3 % of all sales in 2013, up from 94.5 % in 2004) than in Vietnam (82.6 % of all sales in 2013, up from 74.0 % in 2004). Within the Philippines, Coca-Cola is the dominant player accounting for 72.1 % of all sales in 2013 (a slight drop from 74.2 % in 2004); PepsiCo is a distant second, with 14.3 % of sales in 2013 (relatively unchanged from 14.4 % in 2004). Canadian company Cott Corp saw a notable increase to 11.9 % of sales in 2013 (up from 5.9 % in 2004) ostensibly drawn from sales previously captured by the *other* category, which dropped from 5.4 to 1.5 % over this period. Domestic brand Zest-O-Corp holds a minuscule percentage of the market, growing from 0.1 % in 2004 to 0.3 % in 2013.

PepsiCo and Coca-Cola are in closer competition in Vietnam, holding respectively 40.1 % and 36.8 % of all sales in 2013, a small change from 37.4 % and 35.0 % in 2004. Vietnamese domestic companies, Chuong Duong Beverages JSC and Saigon Beverages JSC, which combined held between 13 % and 21 % of the market share from 2004 to 2012, folded after 2012. A new domestic company Saigon Alcohol Beer and Beverages Corp appeared on the market in 2013, although it accounts for only 7.8 % of the market share. A portion of this forfeited market appears to have been captured by PepsiCo, as well as Chinese company Uni-President Enterprises Corp (now holding 4.4 % of market share) and Peruvian company Aje Group (with 1.2 % of market share). The *other* category, while in flux during this period, held 9.4 % of market share in both 2004 and 2013.

### Contribution to added sugar in the Vietnamese diet

Over the intervention period per capita sales of SSCBs rose by 2 l annually in Vietnam. Nutrition information provided by Coca-Cola,^1^ which distributes the top selling SSCB in Vietnam (Coca-Cola, 22 % of market share) reports 39 g of sugar in 12 fluid ounces. Thus 2 l of Coca-Cola would potentially introduce approximately 220 g of added caloric sugar per capita per year into the Vietnamese diet wholly from SSCBs. This is not a dramatic increase, although Euromonitor predicts that consumption will rise by another 7 l per capita per year by 2019, which could introduce another 770 g of added sugar. Moreover, SSCBs are not the only product introducing increased availability of sugar in the beverage market, in Vietnam one of the fastest growing soft drink sectors are ready to drink teas with per capita sales rising from 0.2 l annually in 2000 to 9 l in 2013, almost double carbonate sales, making this another important area to watch for dietary change.

## Discussion

Our analyses revealed two main findings. First, in the year after Vietnam opened its markets to foreign companies, there was a significant increase in sales of SSCBs that was not seen in the control country, Philippines, or in other food sectors we would expect to be unaffected, i.e., unprocessed foods. Second, the main beneficiaries of this growth were foreign beverage companies, namely Coca-Cola and PepsiCo, while domestic beverage companies lost market share. These findings together provide substantial support for the link between trade and investment liberalization and changing food environments characterized by increasingly dominant foreign multinational companies and their archetypal unhealthy dietary products, specifically SSCBs.

Worth noting is that the Philippines, which joined the WTO upon its inception in 1995, had a much larger domestic SSCB market relative to Vietnam. This is consistent with previous findings that trade relations, particularly those with the US, lead to changes in food imports and exports that result in foreign food environments more closely mirroring those in the US [17]. Vietnam had delayed trade relations with both the US and the WTO, which may explain its relatively small, albeit rapidly growing, SSCB market. The data revealed that the Philippines has been experiencing declines in SSCB sales over the years, which can potentially be explained by an emergence of healthy behaviours after the recognition of an expansion in degenerative diseases due to unhealthy dietary patterns [43]. There is no guarantee that Vietnam will reach the level of consumption of countries like the Philippines or the US, in fact, with the advent of globalization and an increased awareness of global health concerns, Vietnam has the opportunity to capitalize on healthy eating trends to mitigate the development of a noncommunicable disease epidemic at a much earlier stage.

Our results are also consistent with market reports from Coca-Cola and PepsiCo. In 2012 Coca-Cola announced that they would invest US $300 million into Vietnam, bringing their total investment up to US $500 million since 2010 [44]. The supply chain manager for Coca-Cola Vietnam remarked that growth has been very fast since 2009 and that their facilities have struggled to meet the demand. Coca-Cola has made investments in its existing plants to maximize production, increasing hourly output from 24,000 bottles to 28,000 bottles in Ho Chi Minh City, and from 30,000 bottles to 35,500 bottles in Hanoi [45]. The company has also invested in new cold-drink coolers to improve sales in local retailers [44]. PepsiCo announced a new investment of US $250 million into Vietnam as of 2011 [46], and has opened three new facilities since 2009, a number equal to what it had opened since entering Vietnam in 1994 [47, 48]. The new facilities include one that was announced to be the largest food and beverage production plant in Asia [46]. Prior to these investments, in the first approximately 16 years that both companies operated in Vietnam, Coca-Cola had invested less than US $150 million and PepsiCo around US $250 million, amounts equal to, or considerably less (in the case of Coca-Cola) than what they have committed in just the past few years [46, 49]. Announcements of investments into the Philippines have been sparser. In 2013 Coca-Cola announced a commitment to put US $1 billion into the Philippines over a 5 year period, where their original investment was considerably larger, with 22 plants to maintain [50]. This came in the same month that Coca-Cola announced it would be moving its concentration plant operations from the Philippines to Singapore, cited as a need to improve efficiencies [51]. There was an announcement from PepsiCo that they would be investing PH $650 million (approximately US $14.5 million) into the Philippines, although this was limited to their snack foods brands, rather than an investment in beverage manufacturing [52].

Our key findings, namely the growth of Vietnam’s SSCB market captured chiefly by foreign companies after FDI liberalization, have important implications for the current TPP negotiations. Vietnam as a promising emerging market will continue to be a prime target for foreign investors looking for growth rates no longer seen in developed countries. Although the data presented in this paper are limited to carbonated beverages sold in retail stores, Vietnam is expected to see further development of their consumer foodservices sector, particularly with leading fast food chains, including KFC, Lotteria, and Jollibee, with whom both PepsiCo Vietnam and Coca-Cola Beverages Vietnam Co Ltd have been collaborating. Fountain sales of soft drinks are forecasted to see increased growth in the next few years making this an important area to watch for increased sales and consumption of SSCBs and an important area for future research [53].

Returning to our concern with the TPP agreement and its inclusion of ISDS provisions, Vietnam has already experienced the so-termed *regulatory chill* associated with trade and investment agreements, and particularly those with ISDS mechanisms. Regulatory chill occurs when a government alters, delays, or abandons regulatory reform out of concern of a trade or investment dispute. A recent attempt by the Vietnamese government to introduce an excise tax on carbonated soft drinks on the grounds that they posed a health risk, was abandoned in July 2014 just months after the American Chamber of Commerce, representing American carbonated beverage companies, released their response stating that “There is a possibility that the tax could be found by international trade bodies to violate Vietnam’s free trade agreements, and it will certainly erode foreign investors’ confidence in Vietnam’s commitment to the national treatment principle [54].” If the Vietnamese government decides to ratify the TPP with its ISDS mechanism in what is purported to be the most comprehensive agreement to date with nations such as the US, which are economically stronger and have considerably more experience in utilizing ISDS provisions, it should do so fully aware of the financial and regulatory repercussions to which they are opening themselves up.

A randomized controlled trial of national trade policy and population dietary outcomes would be inconceivable, thus we made constructive use of naturally occurring conditions in Vietnam and the Philippines to help estimate such effects. Natural experiments can yield valuable evidence where it would be otherwise unattainable. Future analyses of this nature could be strengthened by excluding alternative explanations, including a wider range of falsification tests, or the use of a synthetic control (a composite of multiple regions), rather than a single control country. Additionally, there may have been one or more significant events that took place in Vietnam that may equally or better explain our findings that were outside of the knowledge and control of the researchers. One potential confounder is the parallel introduction of bilateral commitments through the US-Vietnam bilateral agreement, making it difficult to disentangle which specific trade and investment liberalization agreement led to the changes in the SSCB market, in all likelihood both streams of liberalization contributed to growth in this market.

Other factors that may contribute to a country’s investment climate include political and economic stability, infrastructure, wages, corporate tax structures, tax incentives for FDI (including export processing zones) and proximity to main markets (to reduce transport costs) [55, 56]. To our knowledge there were no considerable changes in these factors in Vietnam during our intervention period. Attributing specific patterns in FDI to trade and investment agreements is challenging with even the most sophisticated econometric techniques; this is due in part to the long-term implementation periods of these agreements which make it challenging to capture all FDI activity attributable to the agreement and the difficulty in obtaining disaggregated FDI data due to confidentiality provisions [57]. Deciding where to introduce the time of intervention is also complicated. Our intervention period of 2009, although capturing almost all liberalization we identified as relevant to SSCBs, did not account for the full implementation of commitments incidental to manufacturing, which did not take effect until 1 January 2010. Our findings are limited by the restricted range of data available, particularly after Vietnamese implementation of WTO commitments; the trends in our data are just emerging and will need further analysis with additional data points to validate. Finally, while this analysis focused on Vietnam and the Philippines the intention is that the results will be generalizable to these broader patterns of trade and investment liberalization.

## Conclusions

The current analysis has provided much needed additional quantitative evidence for the link between investment liberalization and changes to the food environment, namely SSCBs. Ongoing efforts to monitor the impacts of trade and investment agreements on food environments [2] will assist in shifting the discourse for action to address the growing burden of diet-related noncommunicable diseases away from individual-oriented strategies to systemic frameworks that recognize structural drivers, including transnational corporations and their supporting neoliberal market liberalization infrastructure. Unifying efforts to build a body of evidence empirically demonstrating the contribution of trade and investment policies to changing food environments and patterns of health outcomes is a first step in being able to make defensible policy decisions to mitigate these impacts.

## Abbreviations

CAFTA: Central America Free Trade Agreement
DID: Difference-in-difference
FDI: Foreign direct investment
GDP: Gross domestic product
NAFTA: North American Free Trade Agreement
SSCBs: Sugar-sweetened carbonated beverages
TPP: Trans-Pacific Partnership
WTO: World Trade Organization

## Footnotes

1 http://productnutrition.thecoca-colacompany.com/
